# Reproducibility and validity of the 3D L.A.S.A.R. posture alignment system in persons with a transtibial bone-anchored prosthesis

**DOI:** 10.1097/PXR.0000000000000497

**Published:** 2025-10-20

**Authors:** Yvonne Witkowski, Niels Jonkergouw, Vera G. M. Kooiman, Ruud A. Leijendekkers

**Affiliations:** 1 Department of Rehabilitation, Radboud University Medical Center, Nijmegen, The Netherlands; 2 Department of Orthopaedic Technology, Military Rehabilitation Centre Aardenburg, Doorn, The Netherlands; 3 Department of Human Movement Sciences, University of Groningen, University Medical Center Groningen, Groningen, The Netherlands

**Keywords:** amputee, alignment, bone-anchored, validity, reproducibility

## Abstract

**Background:**

During the alignment process, the 3D Laser Assisted Static Alignment Reference (L.A.S.A.R.) is clinically used to objectively aid rehabilitation teams to optimize static prosthetic alignment. Although this device is used routinely within the clinic, its reliability and validity are unknown.

**Objective(s):**

To assess the test–retest reproducibility and concurrent validity of the 3D L.A.S.A.R.

**Study Design:**

Clinimetric study.

**Methods:**

In this cross-sectional study, participants using a unilateral transtibial bone-anchored prosthesis were recruited by convenience sampling. Reproducibility was assessed by calculating reliability (intraclass correlation coefficients [ICC]) and agreement parameters (standard error of measurement, smallest detectable change, and limits of agreement). Concurrent validity was assessed through ICC and limits of agreement.

**Results:**

The reliability parameters (ICC_agreement_) ranged from 0.86 to 0.97, with a standard error of measurement (%) of 6.5%‒29.5% in 2D mode and 5.3%‒23.9% in 3D mode and smallest detectable change (%) of 17.9%‒81.6% in 2D mode and 14.6%‒66.1% in 3D mode with no systematic bias. Concurrent validity (ICC_consistency_) ranged from 0.11 to 0.87, with systematic bias found between both systems in several perspectives.

**Conclusions:**

The 3D L.A.S.A.R. shows very good to excellent test–retest reliability. However, the measurement errors exceeded clinically considerable deviations. Concurrent validity ranged between poor to very good. Further development of the 3D L.A.S.A.R. and research focusing on clinimetric properties such as responsiveness or interrater reliability is needed.

## Introduction

Prosthetic alignment is the three-dimensional spatial orientation of prosthetic components to the residual limb. This is an essential process to optimize prosthetic use for both socket-suspended prostheses (SSP) and bone-anchored prostheses (BAP). Suboptimal prosthetic alignment may induce pain and patient dissatisfaction,^1^ increased metabolic energy cost,^2^ and/or gait asymmetry with subsequent physical complaints from compensatory movement strategies.^3^ This eventually leads to reduced prosthetic use and quality of life.^4,5^ Reliable, valid, and responsive measurement instruments are essential in clinical practice to ensure adequate prosthetic alignment and evaluate alignment adjustments over time. The Laser Assisted Static Alignment Reference (L.A.S.A.R.) system has been used in clinical practice for prosthetic alignment for decades. Static alignment references have been established for SSP users, partly based on healthy subjects.^6,7^ For BAP users, reference values are lacking, but some exploratory work is done. However, these (reference) values are hard to interpret as the measurement properties of the used devices are unclear. In the absence of this information, the internal validity of the published results is compromised, as the extent of variability originating from the system itself remains unknown.

Within the alignment process, the 3D L.A.S.A.R. is used for evaluating the static prosthetic alignment.^6,7^ Hereby, a line is virtually plotted on an image of the prosthetic user with the possibility to switch between the Legacy mode, hereafter referred to as “2D mode,” and 3D mode. Despite the extensive usage, it lacks published clinimetric properties, raising questions about its value in clinical practice compared with gait analysis systems, such as Vicon. Vicon is an optical motion system that obtains highly accurate static and dynamic kinematic data and was proven to have high test–retest reproducibility.^8^ However, the high cost, extensive time requirements, and need for in-depth technical expertise limit its use outside laboratory settings.^9^

To investigate the clinical value of the 3D L.A.S.A.R., the primary research question was: What is the test–retest reproducibility and concurrent validity of 2D and 3D mode static alignment measurements on the 3D L.A.S.A.R. in the frontal and sagittal plane in patients with transtibial BAP? With a secondary question on: What is the reproducibility and validity within the transverse plane?

## Methods

The study protocol was approved by the Medical Research Ethics Committee Oost-Nederland (NL78861.091.22) and all participants provided written informed consent before the start of the study. The study was conducted according to the Declaration of Helsinki^10^ and COSMIN reporting standards.^11^

### Participants

COSMIN proposes a minimal sample size of n = 50 to draw statistically valid conclusions for reliability and validity studies.^12,13^ Therefore, it was striven *a priori* to include 50 participants for this cross-sectional clinimetric study. The study population was recruited using a convenience sampling strategy within the Radboud University Medical Center (RUMC) (Nijmegen, the Netherlands) and Military Rehabilitation Centre Aardenburg (Doorn, the Netherlands).

Participants eligible for inclusion had a unilateral transtibial BAP and could stand freely without walking aids. Potential subjects unable to load the prosthetic leg because of balance disorders, musculoskeletal injuries in the lower extremity, or pain were excluded.

### Equipment

#### 3D L.A.S.A.R

The 3D L.A.S.A.R. (L.A.S.A.R. Posture, Ottobock HealthCare GmbH, Duderstadt, Germany) consists of embedded force plates and cameras in the frontal and sagittal planes. The derivative of the obtained ground reaction force (GRF) for each leg is digitally visualized (on a tablet) as “load lines” on an image of the patient.^7^ The 2D mode shows 2 vertically orientated red load lines (GRF_vertical_), indicating the point of application.^14^ The 3D mode shows a blue and a green vector line (GRF_vector_),^7^ displaying the point of application and direction of the ground reaction force vector.^14^ In both the 2D and 3D modes, the magnitude of a ground reaction force can be derived from the weight distribution on the legs. These forces in newton and the torsion moments within the transverse plane in newton meters are displayed at the bottom corners of the screen. A negative value indicated an external and a positive value an internal moment.

#### M-gait

The M-Gait's embedded force plates (Motek Medical, NL, 2000 Hz) were used to obtain the required GRF data. This was combined with the real-time (infrared) camera system (Vicon) tracking retroreflective markers, enabling 3D visualization of the GRF_vector_ with a point of application, direction, and magnitude.^15^

### Study design

Two cohorts were included, of which cohort 1 was enrolled prospectively from June 2022 till February 2023 at RUMC, and data were collected using a borrowed 3D L.A.S.A.R. device from Ottobock. Cohort 2 enrolled participants prospectively from February till June 2024 to reach the predetermined sample size, and data were collected with 2 additional 3D L.A.S.A.R. devices (Figure 1). An accuracy protocol was set up to test for systematic error between the 3 3D L.A.S.A.R. devices used (Supplemental Digital Content 1, http://links.lww.com/POI/A342).

**Figure 1.**
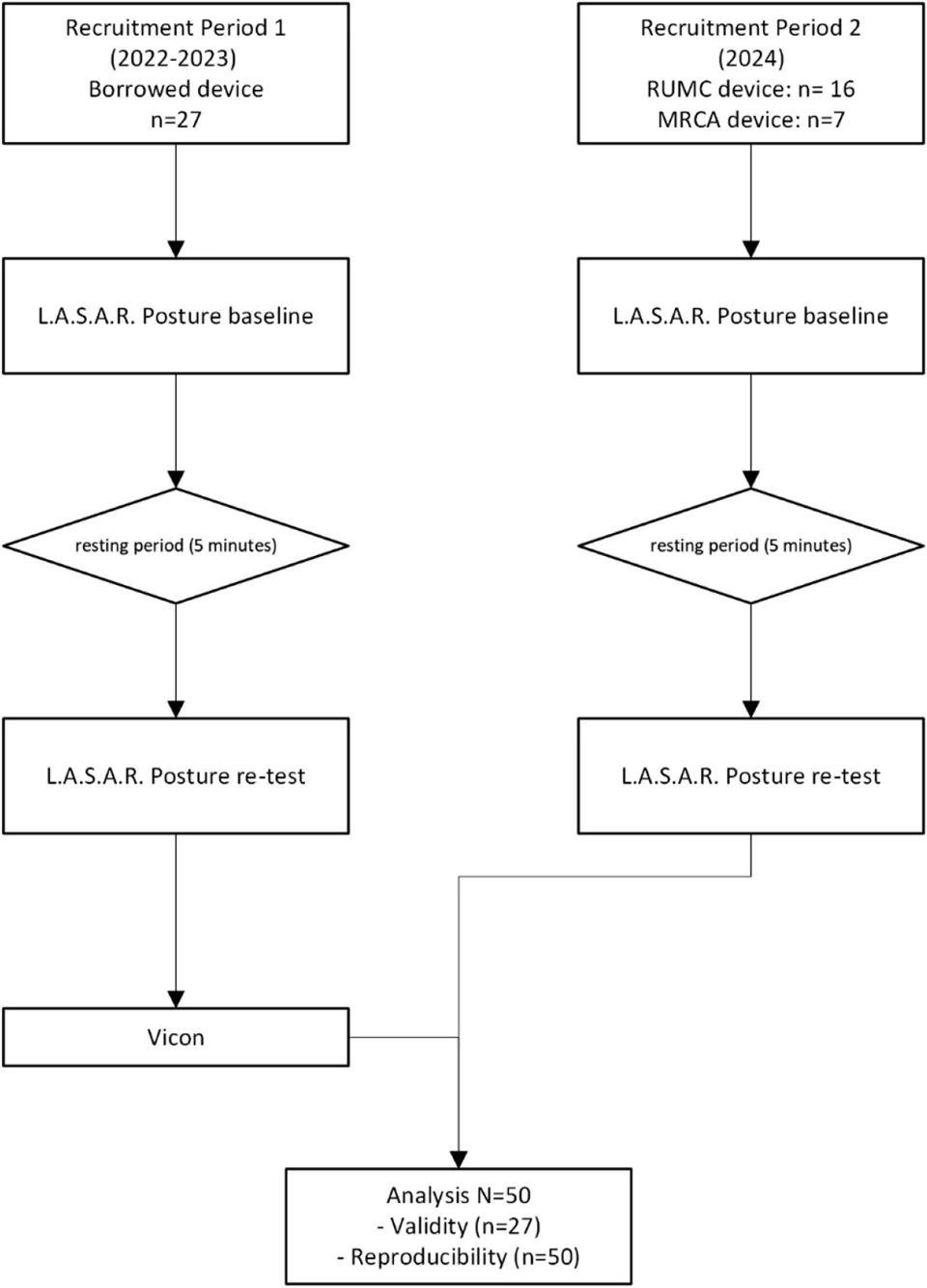
Study flowchart.

All data collection was conducted in one session, beginning with recording demographic data (sex, age, height, weight, stump girth/length, affected side, type/size prosthetic foot, type connector, reason for amputation, and date of initial/osseointegration surgery).

A retroreflective marker was placed on each leg on the lateral knee following the Vicon Plug-in gait model.^16^ After the calibration of the 3D L.A.S.A.R. in both planes by equipment guidelines,^17^ participants were asked to obtain a standardized standing position (relaxed stance, with their arms alongside their body, looking forward) on the 3D L.A.S.A.R. while wearing their own prosthesis and habitual footwear.^7^ A 10 cm-wide block was manually held between the femoral epicondyles to achieve a standardized stance width and removed after confirming that no active knee adduction was performed to hold the block between both knees. Both feet were placed in 5 degree external rotation using a foot orientation paper and stood at the same level in anterior–posterior direction on the device (Figure 2). Equal limb loading was controlled and verbally corrected to obtain balanced weight bearing. No other feedback was provided to guarantee independent measures.

**Figure 2.(:**
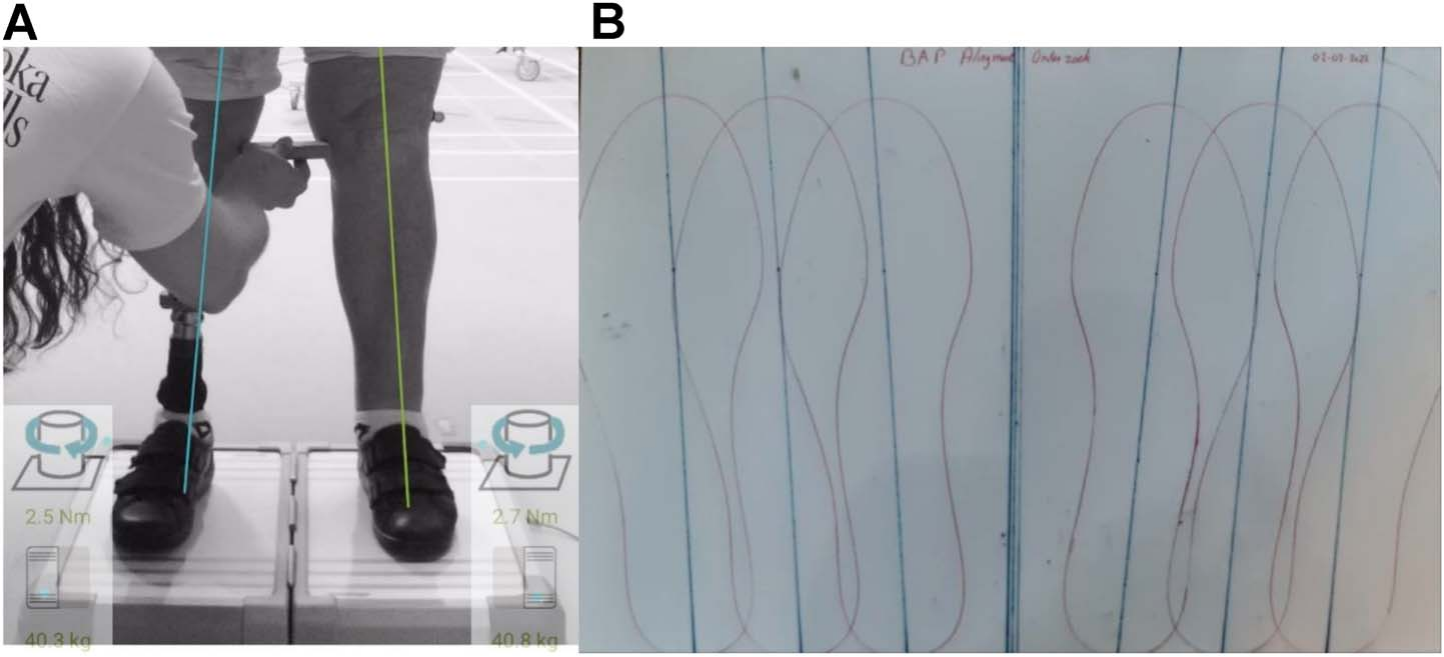
a) Setup during the testing procedure to standardize the stancewidth and foot orientation, and (b) the foot orientation paper.

At least a five-minute test–retest interval was used^18^ without detaching the prosthesis or markers between the measurements. Each 3D L.A.S.A.R. measurement included 8 photographs per participant, comprising 4 photographs from the sagittal plane measuring both legs in 2D and 3D modes, and 4 photographs from the frontal plane, with photographs measuring both legs from the dorsal and ventral perspectives in 2D and 3D modes.

Cohort 1 underwent additional Vicon measurements for validity assessments at RUMC after the 3D L.A.S.A.R (Figure 1), following the same foot placement and standing position protocol. Participants stood on the embedded force plates for one minute.

### Data analysis

3D L.A.S.A.R. photographs were analyzed using the integrated software's graphical reference points. In the frontal plane, the horizontal distance from the load line to the base of the marker was measured. In the sagittal plane, the horizontal distance between the load line and the knee axis (middle of the marker) was measured using a visual gauge (Figure 3).

**Figure 3.**
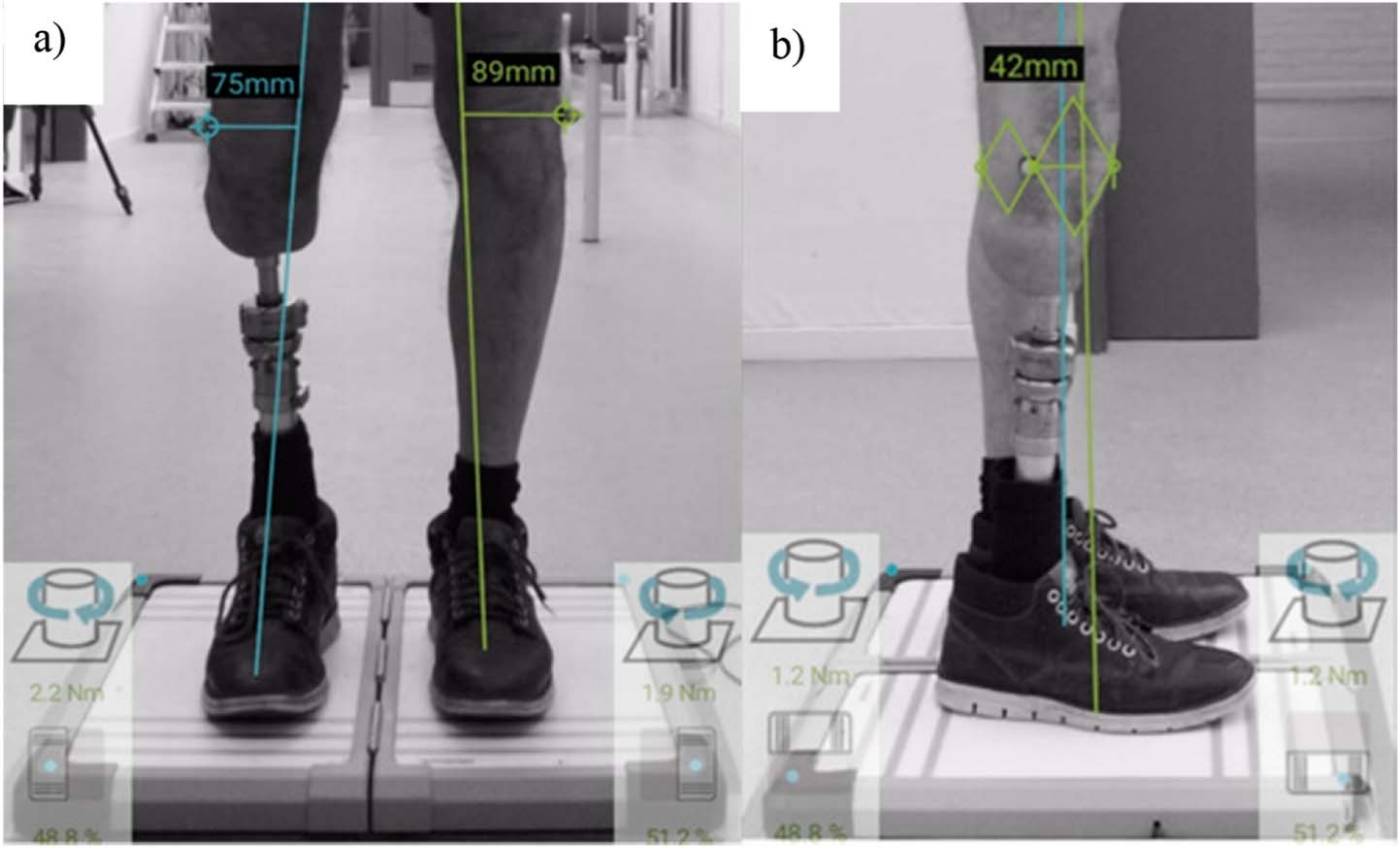
Examples of the measurements taken with the L.A.S.A.R. posture of the horizontal deviation in the (a) frontal plane (3D mode) and (b) sagittal plane (3D mode).

Raw data of the Vicon measurements (Figure 4) were preprocessed and filtered (Woltring filter, Mean Square Error = 10) in Nexus 2.10. The GRF data were filtered (zero-lag 10 Hz, low-pass fourth-order Butterworth filter), and further data analyses were performed with MATLAB 2019b (The MathWorks Inc, Natick, Massachusetts, United States). The mean horizontal distance from the GRF_vector_ until the middle of the marker in the frontal and sagittal planes were calculated based on all data from the one-minute stance.

**Figure 4.**
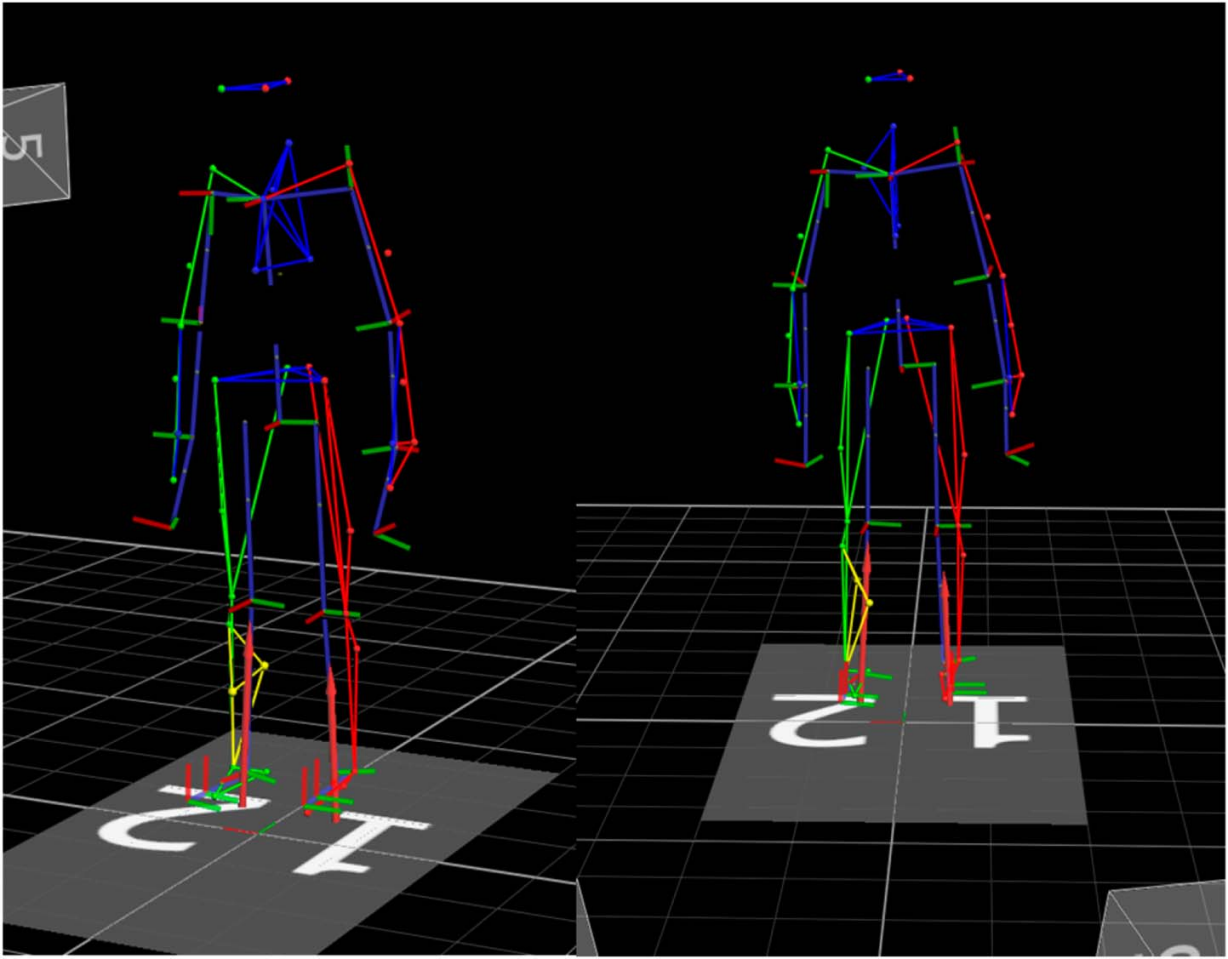
Raw data of Vicon measurements; the red arrow originating from the center of pressure in the foot shows the ground reaction force vector. The balls represent the retroreflective markers that were put according to the Vicon Plug-in-Gait model.

### Statistical analysis

Continuous demographic and descriptive data were reported on the total cohort using the mean and standard deviation (SD), or the median plus 25th and 75th percentiles based on their normality distribution. Frequencies and percentages were used for categorical data.

### Reproducibility

The construct reproducibility was split up in reliability parameters and agreement parameters. All reproducibility parameters were calculated with the data of the test–retest measurements and structured by mode (2D, 3D), plane (frontal, sagittal), perspective (dorsal, ventral), and leg (prosthetic, intact). Intraclass correlation coefficients (ICC) with 95% confidence interval (CI) were calculated, using a single-measure absolute-agreement, 2-way, mixed-effect model (ICC(3, 1)_agreement_).^19^

Agreement parameters were determined by calculating the standard error of measurement (SEM_agreement_) and smallest detectable change (SDC_agreement_), expressed in millimeters and percentages.^20^ The variance components for the SEM_agreement_ calculation were obtained through a VARCOMP analysis (MINQUE model). The exact formulas used were: SEM_agreement_ = √σ^2^_error_ = √(σ^2^_observer_ + σ^2^_residual_), and SDC_agreement_ = 1.96*√2*SEM_agreement_.^12,20^ In addition, the percentages of the mean measurements (%SEM_agreement_ and %SDC_agreement_, respectively) were determined to be able to make generalized conclusions. The %SEM_agreement_ and %SDC_agreement_ were calculated through dividing the respective SEM_agreement_ or SDC_agreement_ by the average of the test and the retest measurements and then multiplying it by 100.^21^ Possible systematic bias in measurement error was visualized through Bland and Altman plots, including 95% limits of agreement (LoA) calculated as: mean_difference_ ± 1.96*SD_difference_.^22^

### Validity

Concurrent validity between the 3D L.A.S.A.R. and Vicon was analyzed by calculating ICC and 95% CI based on a single-measure, consistency, 2-way, mixed-effect model (ICC(3, 1)_consistency_).^19^ Possible systematic bias in measurement error was visualized through Bland and Altman plots, including 95% LoA.^22^ As the 3D L.A.S.A.R. force plates were found to produce bias (Supplemental Digital Content 1, http://links.lww.com/POI/A342), the analyses were structured by mode, plane, leg, and force plate (right, left).

The interpretation of all ICC estimates was: 0.01‒0.20 poor, 0.21‒0.40 slight, 0.41‒0.60 fair, 0.61‒0.80 good, 0.81‒0.92 very good, and 0.93‒1.00 excellent reproducibility or validity.^23^ IBM SPSS Statistics v29 (IBM SPSS, Chicago, Illinois, United States) was used for all statistical analyses. A two-sided *p* < 0.05 was considered statistically significant in all calculations. The same analyses and interpretation were used to analyze the data collected in the transverse plane. The methodology and results for the secondary research question are found in Supplemental Digital Content 2, http://links.lww.com/POI/A343.

## Results

### Participant characteristics

The total study population consisted of 50 participants (cohort 1 = 27, cohort 2 = 23). The mean age was 55.5 ± 12.6 years, and the mean height was 177.0 ± 10.5 cm, with an adjusted BMI^24^ of 28.9 (27.1; 32.9) kg/m^2^. Details of both cohorts are displayed in Table 1.

**Table 1.**
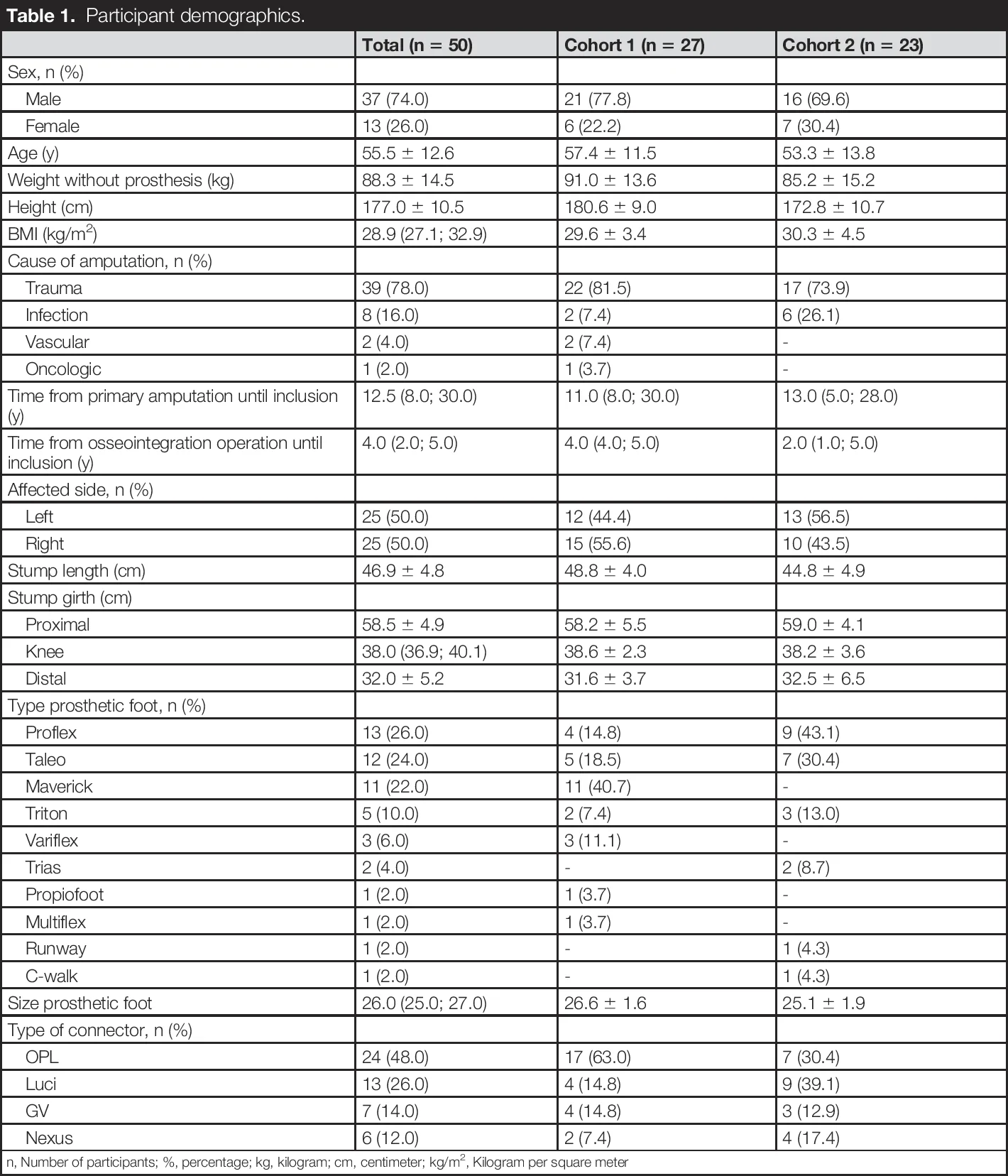
Participant demographics.

	Total (n = 50)	Cohort 1 (n = 27)	Cohort 2 (n = 23)
Sex, n (%)			
Male	37 (74.0)	21 (77.8)	16 (69.6)
Female	13 (26.0)	6 (22.2)	7 (30.4)
Age (y)	55.5 ± 12.6	57.4 ± 11.5	53.3 ± 13.8
Weight without prosthesis (kg)	88.3 ± 14.5	91.0 ± 13.6	85.2 ± 15.2
Height (cm)	177.0 ± 10.5	180.6 ± 9.0	172.8 ± 10.7
BMI (kg/m^2^)	28.9 (27.1; 32.9)	29.6 ± 3.4	30.3 ± 4.5
Cause of amputation, n (%)			
Trauma	39 (78.0)	22 (81.5)	17 (73.9)
Infection	8 (16.0)	2 (7.4)	6 (26.1)
Vascular	2 (4.0)	2 (7.4)	-
Oncologic	1 (2.0)	1 (3.7)	-
Time from primary amputation until inclusion (y)	12.5 (8.0; 30.0)	11.0 (8.0; 30.0)	13.0 (5.0; 28.0)
Time from osseointegration operation until inclusion (y)	4.0 (2.0; 5.0)	4.0 (4.0; 5.0)	2.0 (1.0; 5.0)
Affected side, n (%)			
Left	25 (50.0)	12 (44.4)	13 (56.5)
Right	25 (50.0)	15 (55.6)	10 (43.5)
Stump length (cm)	46.9 ± 4.8	48.8 ± 4.0	44.8 ± 4.9
Stump girth (cm)			
Proximal	58.5 ± 4.9	58.2 ± 5.5	59.0 ± 4.1
Knee	38.0 (36.9; 40.1)	38.6 ± 2.3	38.2 ± 3.6
Distal	32.0 ± 5.2	31.6 ± 3.7	32.5 ± 6.5
Type prosthetic foot, n (%)			
Proflex	13 (26.0)	4 (14.8)	9 (43.1)
Taleo	12 (24.0)	5 (18.5)	7 (30.4)
Maverick	11 (22.0)	11 (40.7)	-
Triton	5 (10.0)	2 (7.4)	3 (13.0)
Variflex	3 (6.0)	3 (11.1)	-
Trias	2 (4.0)	-	2 (8.7)
Propiofoot	1 (2.0)	1 (3.7)	-
Multiflex	1 (2.0)	1 (3.7)	-
Runway	1 (2.0)	-	1 (4.3)
C-walk	1 (2.0)	-	1 (4.3)
Size prosthetic foot	26.0 (25.0; 27.0)	26.6 ± 1.6	25.1 ± 1.9
Type of connector, n (%)			
OPL	24 (48.0)	17 (63.0)	7 (30.4)
Luci	13 (26.0)	4 (14.8)	9 (39.1)
GV	7 (14.0)	4 (14.8)	3 (12.9)
Nexus	6 (12.0)	2 (7.4)	4 (17.4)

n, Number of participants; %, percentage; kg, kilogram; cm, centimeter; kg/m^2^, Kilogram per square meter

### Reproducibility

Overall, very good to excellent reliability was determined based on the 12 ICC (3, 1)_agreement_ (Table 2). Within the 2D mode, the ICC (3, 1)_agreement_ ranged from 0.86 to 0.97, and in 3D mode from 0.88 to 0.95.

**Table 2.**
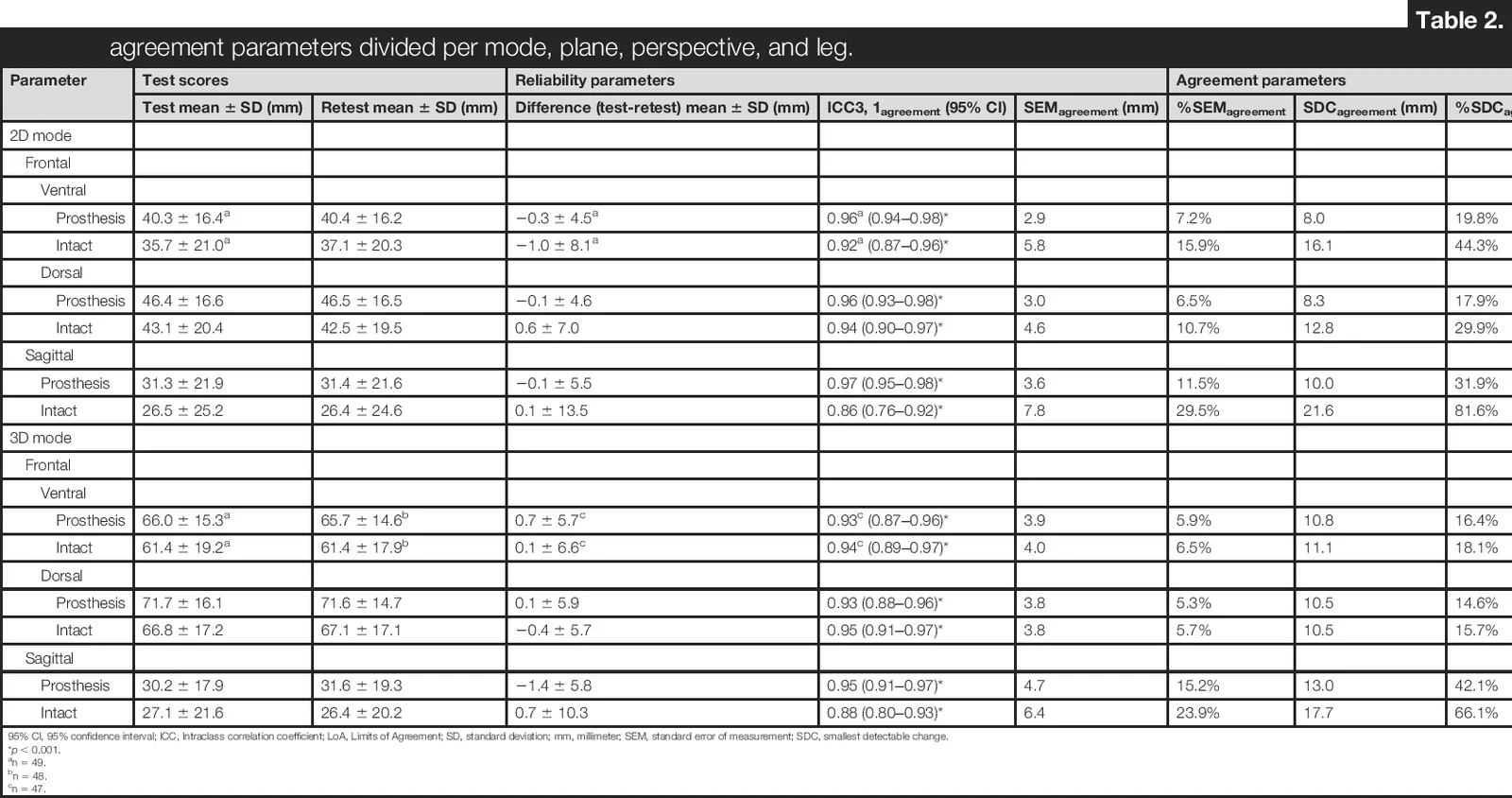
Reliability and agreement parameters divided per mode, plane, perspective, and leg.

Parameter	Test scores		Reliability parameters			Agreement parameters			
	Test mean ± SD (mm)	Retest mean ± SD (mm)	Difference (test-retest) mean ± SD (mm)	ICC3, 1_agreement_ (95% CI)	SEM_agreement_ (mm)	%SEM_agreement_	SDC_agreement_ (mm)	%SDC_agreement_	LoA 95% CI (mm)
2D mode									
Frontal									
Ventral									
Prosthesis	40.3 ± 16.4^a^	40.4 ± 16.2	−0.3 ± 4.5^a^	0.96^a^ (0.94‒0.98)*	2.9	7.2%	8.0	19.8%	−9.1 to 8.5
Intact	35.7 ± 21.0^a^	37.1 ± 20.3	−1.0 ± 8.1^a^	0.92^a^ (0.87‒0.96)*	5.8	15.9%	16.1	44.3%	−16.9 to 14.9
Dorsal									
Prosthesis	46.4 ± 16.6	46.5 ± 16.5	−0.1 ± 4.6	0.96 (0.93‒0.98)*	3.0	6.5%	8.3	17.9%	−9.1 to 8.9
Intact	43.1 ± 20.4	42.5 ± 19.5	0.6 ± 7.0	0.94 (0.90‒0.97)*	4.6	10.7%	12.8	29.9%	−13.1 to 14.3
Sagittal									
Prosthesis	31.3 ± 21.9	31.4 ± 21.6	−0.1 ± 5.5	0.97 (0.95‒0.98)*	3.6	11.5%	10.0	31.9%	−10.9 to 10.7
Intact	26.5 ± 25.2	26.4 ± 24.6	0.1 ± 13.5	0.86 (0.76‒0.92)*	7.8	29.5%	21.6	81.6%	−26.3 to 26.6
3D mode									
Frontal									
Ventral									
Prosthesis	66.0 ± 15.3^a^	65.7 ± 14.6^b^	0.7 ± 5.7^c^	0.93^c^ (0.87‒0.96)*	3.9	5.9%	10.8	16.4%	−10.5 to 11.9
Intact	61.4 ± 19.2^a^	61.4 ± 17.9^b^	0.1 ± 6.6^c^	0.94^c^ (0.89‒0.97)*	4.0	6.5%	11.1	18.1%	−12.8 to 13.0
Dorsal									
Prosthesis	71.7 ± 16.1	71.6 ± 14.7	0.1 ± 5.9	0.93 (0.88‒0.96)*	3.8	5.3%	10.5	14.6%	−11.5 to 11.7
Intact	66.8 ± 17.2	67.1 ± 17.1	−0.4 ± 5.7	0.95 (0.91‒0.97)*	3.8	5.7%	10.5	15.7%	−11.6 to 10.8
Sagittal									
Prosthesis	30.2 ± 17.9	31.6 ± 19.3	−1.4 ± 5.8	0.95 (0.91‒0.97)*	4.7	15.2%	13.0	42.1%	−12.8 to 10.0
Intact	27.1 ± 21.6	26.4 ± 20.2	0.7 ± 10.3	0.88 (0.80‒0.93)*	6.4	23.9%	17.7	66.1%	−19.5 to 20.9

95% CI, 95% confidence interval; ICC, Intraclass correlation coefficient; LoA, Limits of Agreement; SD, standard deviation; mm, millimeter; SEM, standard error of measurement; SDC, smallest detectable change.

*p < 0.001.

a n = 49.

b n = 48.

c n = 47.

Within the 2D mode, the %SEM_agreement_ varied between 6.5% and 15.9% in the frontal plane and 11.5%‒29.5% in the sagittal plane. The %SDC_agreement_ varied between 17.9%‒44.3% in the frontal plane and 31.9%‒81.6% in the sagittal plane. The LoA were smaller in the prosthetic leg compared with the intact leg from both the dorsal and ventral perspectives in the frontal and in the sagittal planes.

Within the 3D mode, the %SEM_agreement_ varied between 5.3%‒6.5% in the frontal plane and 15.2%‒23.9% in the sagittal plane. The %SDC_agreement_ varied between 14.6% and 18.1% in the frontal plane and 42.1%‒66.1% in the sagittal plane. The LoA were smaller in the prosthetic leg compared with the intact leg from both the dorsal and ventral perspectives in the frontal and in the sagittal planes. Detailed information is found in Supplemental Digital Content 3, http://links.lww.com/POI/A344.

### Validity

Within the 2D mode, fair to good validity was found in the frontal plane, with ICC (3, 1)_consistency_ varying between 0.57 and 0.78. In the sagittal plane, the validity was poor to slight with ICC (3, 1)_consistency_ varying between 0.17 and 0.30.

Within the 3D mode, good to very good validity was found in the frontal plane, with ICC (3, 1)_consistency_ varying between 0.67 and 0.87. In the sagittal plane, the validity was poor to fair with ICC (3, 1)_consistency_ varying between 0.11 and 0.41.

The Bland and Altman plots demonstrated that agreement was consistent over the measurement range for all analyses. Despite that, systematic bias in measurement error was detected in several perspectives (Table 3, Supplemental Digital Content 4, http://links.lww.com/POI/A345). The LoA had a narrower range for the right force plate compared with the left in the frontal plane in both legs and mode.

**Table 3.**
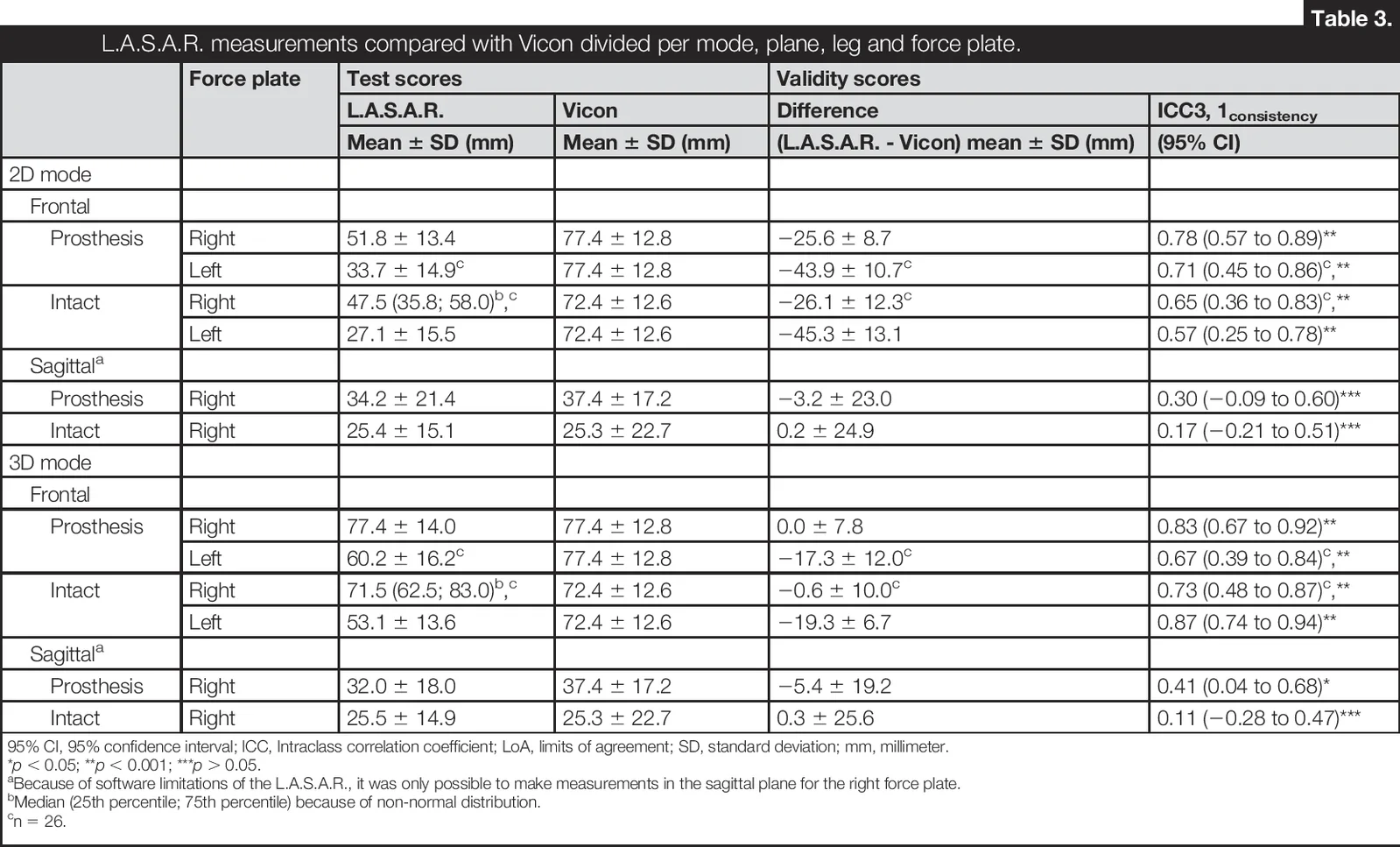
Validity of the L.A.S.A.R. measurements compared with Vicon divided per mode, plane, leg and force plate.

	Force plate	Test scores		Validity scores		
		L.A.S.A.R.	Vicon	Difference	ICC3, 1_consistency_	LoA
		Mean ± SD (mm)	Mean ± SD (mm)	(L.A.S.A.R. - Vicon) mean ± SD (mm)	(95% CI)	95% CI (mm)
2D mode						
Frontal						
Prosthesis	Right	51.8 ± 13.4	77.4 ± 12.8	−25.6 ± 8.7	0.78 (0.57 to 0.89)**	−42.7 to −8.5
	Left	33.7 ± 14.9^c^	77.4 ± 12.8	−43.9 ± 10.7^c^	0.71 (0.45 to 0.86)^c,^**	−64.9 to −22.9
Intact	Right	47.5 (35.8; 58.0)^b^,^c^	72.4 ± 12.6	−26.1 ± 12.3^c^	0.65 (0.36 to 0.83)^c,^**	−50.2 to −2.0
	Left	27.1 ± 15.5	72.4 ± 12.6	−45.3 ± 13.1	0.57 (0.25 to 0.78)**	−71.0 to −19.6
Sagittal^a^						
Prosthesis	Right	34.2 ± 21.4	37.4 ± 17.2	−3.2 ± 23.0	0.30 (−0.09 to 0.60)***	−48.3 to 41.9
Intact	Right	25.4 ± 15.1	25.3 ± 22.7	0.2 ± 24.9	0.17 (−0.21 to 0.51)***	−48.6 to 49.0
3D mode						
Frontal						
Prosthesis	Right	77.4 ± 14.0	77.4 ± 12.8	0.0 ± 7.8	0.83 (0.67 to 0.92)**	−15.3 to 15.3
	Left	60.2 ± 16.2^c^	77.4 ± 12.8	−17.3 ± 12.0^c^	0.67 (0.39 to 0.84)^c,^**	−40.8 to 6.2
Intact	Right	71.5 (62.5; 83.0)^b^,^c^	72.4 ± 12.6	−0.6 ± 10.0^c^	0.73 (0.48 to 0.87)^c,^**	−20.2 to 19.0
	Left	53.1 ± 13.6	72.4 ± 12.6	−19.3 ± 6.7	0.87 (0.74 to 0.94)**	−32.4 to −6.2
Sagittal^a^						
Prosthesis	Right	32.0 ± 18.0	37.4 ± 17.2	−5.4 ± 19.2	0.41 (0.04 to 0.68)*	−43.0 to 32.2
Intact	Right	25.5 ± 14.9	25.3 ± 22.7	0.3 ± 25.6	0.11 (−0.28 to 0.47)***	−49.9 to 50.5

95% CI, 95% confidence interval; ICC, Intraclass correlation coefficient; LoA, limits of agreement; SD, standard deviation; mm, millimeter.

*p < 0.05; **p < 0.001; ***p > 0.05.

a Because of software limitations of the L.A.S.A.R., it was only possible to make measurements in the sagittal plane for the right force plate.

b Median (25th percentile; 75th percentile) because of non-normal distribution.

c n = 26.

## Discussion

In this study, the reliability and validity of the 3D L.A.S.A.R. was determined through comparison with Vicon. Test–retest reliability was found to be very good to excellent in all modes, planes, perspectives, and legs, indicating good distinguishability between patients. However, the agreement parameters demonstrated that the 3D L.A.S.A.R. devices did not produce accurate measurements. As the 3D L.A.S.A.R. is used for evaluation purposes, the measurement error becomes more important than the distinguishability.^25^ Measurement errors were found to be at least 8.0 mm (2D mode) and 10.5 mm (3D mode) in the frontal plane for the prosthetic leg, whereas translatory modifications in prosthetic alignment of 1‒2 mm can lead to a change in the perceived fit from painful to comfortable. Ideally, the measurement error is smaller than a clinically relevant change to be able to differentiate true changes from measurement error when performing minor prosthetic adjustments; however, this is not the case.

When evaluating concurrent validity, the ICC in the frontal plane were higher in 3D mode compared with 2D mode for both legs, except for the prosthetic leg standing on the left 3D L.A.S.A.R. force plate. In the sagittal plane, the ICC of the prosthetic leg was higher in 3D mode compared with 2D mode, whereas for the intact leg, the 2D mode scored higher. Compared with Vicon, which has a static positioning error of 0.15 mm,^8^ average positioning differences and therefore validity of the 3D L.A.S.A.R. seems to be dependent from the accuracy of the embedded force plates. As we have observed in our accuracy measurements (Supplemental Digital Content 1, http://links.lww.com/POI/A342), each force plate per device had a different static positioning error. The right force plate had an average deviation in the frontal plane (3D mode) between 0 and 0.6 mm, while the left ranged between 17.0 and 19.0 mm. Measurements from the transverse plane showed similar patterns for both reproducibility and validity (Supplemental Digital Content 2, http://links.lww.com/POI/A343) but performed worse overall. This raises the question about the usability of the frontal and sagittal plane measurements, because changes of transverse plane measurements affect these planes.^26^

Our results show proportionally higher agreement parameters in the sagittal plane compared with the frontal plane, with the intact leg showing the lowest ICCs and highest SEM or SDC within the sagittal plane in both modes. This could originate in the varying compensation strategies to maintain static balance, as patients using a unilateral transtibial prosthesis are able to correct balance perturbations with the intact leg^27,28^ by putting more pressure on the forefoot or rearfoot while maintaining an even weight distribution on both legs.

It is interesting that previous studies using the L.A.S.A.R. described substantially lower averaged distances between anatomical reference points and the load lines in transtibial SSP patients compared with the detected deviations in our BAP-population.^6,7^ The reported results of SSP users are based on very small samples and single measures,^6,7^ and insights in clinimetric properties on SSP users remain unknown. This limits comparability to our findings and suggests that our results should not be extrapolated to the unilateral transtibial SSP population. Nevertheless, the shown clinimetric properties would suggest that these measurement errors would influence clinical measurements on SSP users as well and warrant further research for that particular study population.^6,7,29^ A possible influence for this could be the device-specific variations in deviations between the used 3D L.A.S.A.R. systems found during the accuracy measurement (Supplemental Digital Content 1, http://links.lww.com/POI/A342).

This study exclusively included BAP users, which allows to estimate reliability and agreement parameters without confounding through presence of a pseudojoint as in SSP. Together with the use of a standardized protocol, the use of the same equipment throughout the study, as well as training of raters, this contributed to increase of measurement consistency and minimized measurement errors. The sample size of n = 27 for the validity analyses led to an underpowered statistical analysis, which should be taken into consideration when interpreting the results. There is an increased risk for type II errors, and the ICC estimates can be less precise as presumed from the found results. The use of a convenience sample limits extrapolation of the found results to the broader BAP population. However, as the Dutch BAP population is relatively small, the study population represents approximately 46% of the total, reducing the consequences of the sampling strategy. The accuracy measurement demonstrated systematic measurement error, including some remaining unknown error because of software limitations prohibiting the measurement of horizontal deviations of the distal force plates in the sagittal plane (Supplemental Digital Content 1, http://links.lww.com/POI/A342). This error, together with the use of 3 different 3D L.A.S.A.R. devices, is a limitation of the study's design. Nonetheless, it does highlight the importance for manufacturers to obtain consistent accuracy between devices.

Health professionals mostly use the sagittal plane for assessing static alignment; however, we found that the sagittal plane scored worse than the frontal plane for test–retest reproducibility and validity, despite the efforts to achieve a standardized stance, which is more advanced than usually done in clinical practice. The reported interdevice variance predicts discrepancies between settings in all planes, and using different devices on the same BAP user could therefore lead to false measurements and unnecessary alignment changes.

Thus, clinicians using the 3D L.A.S.A.R. with BAP users are advised to use a more standardized procedure (as described in the “Study Design” section) to minimize additional measurement error and increase precision. While doing so, the 3D mode should be used to take repeated measures of patients in both planes. In the frontal plane, the ventral perspective should be used and changes above SDC% 16.4% (prosthetic leg) or 18.1% (intact leg) indicate true changes. Within the sagittal plane, alignment adjustments above SDC% 42.1% (prosthetic leg) or 66.1% (intact leg) indicate true changes and it is important to compare load line symmetry of the prosthetic to the non-prosthetic leg.

Despite the inaccurate agreement parameters, the 3D L.A.S.A.R. holds clinical value as comparable devices that can capture static alignment in a clinical setting are lacking. In the frontal plane, the static knee moment arm has a considerable association with the dynamic knee moment arm. Thus, in perspective of the prosthetic alignment procedure, static alignment–which is done before dynamic alignment–is fundamental and can therefore be influential for the further alignment process.^30^ The 3D L.A.S.A.R. can help to provide a starting point while standing on the prosthesis. Without this step, only bench alignment is considered, which insufficiently accounts for the prosthesis's weight-bearing dynamics. It remains essential to continue with the objective assessment of dynamic alignment. This step evaluates primary alignment as well as prosthetic functioning. Static alignment is used to assess standing balance and is used as a tool to assist clinical reasoning in respect to alignment adjustments; therefore, it is important to take the detected measurement errors into account. However, as our clinical experience is that even small alignment adjustments (1‒2 mm) can contribute to pain reduction and improved prosthetic comfort and can potentially enhance prosthesis utilization, physical function, and overall quality of life. This would suggest that, clinically, it is understandable that smaller adjustments than the measurement errors could be required and could be made independent on what the 3D L.A.S.A.R. would suggest.

In the future, clinimetric studies with prospective experimental designs should further explore other measurement properties of the 3D L.A.S.A.R. within the BAP or SSP population, also differentiating between amputation levels. A cross-sectional study on interrater reliability could provide information whether patients can be measured by different clinicians, which is a common situation in clinical practice. One could also assess the level of responsiveness for changes in static alignment over time through longitudinal, observational studies, including regular accuracy measurements of the horizontal deviations to monitor whether the direction and magnitude of measurement error remains stable.

## Conclusions

Reproducibility and validity results indicate that the 3D L.A.S.A.R. has very good to excellent test–retest reliability; however, measurement errors exceed clinically relevant deviations. Concurrent validity ranged between poor to very good. Consequently, the utility of the 3D L.A.S.A.R. is questionable and depends on the plane of measurement, leg of interest, and device. Inaccurate measurement instruments have the effect of producing wrong clinical decisions and reduce the quality of care. Therefore, further development of the 3D L.A.S.A.R. devices, on multiple aspects, is essential to guarantee precise measurements.

## Funding

This research received funding from the Military Rehabilitation Centre, Ministry of Defense and the Innovatiefonds Stichting ziektenkosten Verzekering Krijgsmacht (Grandnr.21-038).

Declaration of AI and AI-assisted technologies in the writing process: During the preparation of this work the authors used ChatGPT in order to enhance the wording of this article. After using this tool/service, the authors reviewed and edited the content as needed and take(s) full responsibility for the content of the publication.

## Declaration of conflicting interest

The authors disclosed no potential conflicts of interest with respect to the research, authorship, and/or publication of this article.

## Supplemental material

Supplemental material for this article is available in this article. Direct URL citation appears in the text and is provided in the HTML and PDF versions of this article on the journal's Web site (www.POIjournal.org).

## Supplementary Material
