# Supplementary material for: Reproducibility and validity of the 3D L.A.S.A.R. posture alignment system in persons with a transtibial bone-anchored prosthesis
Source: Prosthet Orthot Int. 2025 Oct 20;50(5):620–9. doi: 10.1097/PXR.0000000000000497 (PMC13645406; doi:10.1097/PXR.0000000000000497)
Supplement: Supplementary file 1 [file poi-50-620-s001.docx]

**Supplemental Digital Content 1 – Accuracy measurements**

During the study, three different 3D L.A.S.A.R. devices were utilized.

To assess the possible measurement error of the devices involved, which can introduce bias in the reliability estimations, a protocol was developed to measure baseline accuracy, which involved the following steps

1. Before placing the weight on the force plate, verify whether the force plates are calibrated in both planes. Reposition the force plates if necessary.
2. One retroreflective marker is fixated with double-sided tape on top of the handle of a two-plated weight stack. It is centered in anteroposterior and mediolateral direction.
3. The weight is placed in the center on one force plate, maintaining the same distance in anteroposterior and mediolateral direction.
4. Photos are taken in the frontal plane, in 2D and 3D mode.
5. Photos are taken in the sagittal plane, in 2D and 3D mode.
6. The weight is displaced from the device
7. The calibration is checked as in step 1.
8. The weight is placed to the center of the other force plate, as in step 3.
9. Photos are taken in the frontal plane, in 2D and 3D mode.
10. Photos are taken in the sagittal plane, in 2D and 3D mode.

In total, eight photos were taken for each device. Afterwards, the horizontal distance from the load line to the middle of the retroreflective marker was measured in millimeters using the 3D L.A.S.A.R. Posture software. The measurements were distinguished at the level of: plane (frontal vs. sagittal), mode (2D vs. 3D), force plate (left vs. right). At all levels the devices were compared to one another. Due to software limitations, the horizontal distance to the load line could not be measured for all three devices of the most distal force plate in the sagittal plane, which leaves an unknown measurement error (Figure 1). An alternative approach to assess the accuracy and validity of the distal (left) force plate could have involved rotating the plate 180 degrees and subsequently repeating steps 1, 2, 3, and 5 of the accuracy measurements and subjecting participants to additional measurements. Implementing such a procedure would have required recalibration and precise repositioning of the force plate after each rotation, significantly increasing measurement time and potential additional errors in respect to recalibration with limited additional benefit for the participant.

It was also found that once the 3D L.A.S.A.R. was rotated 180 degrees relative to the cameras, the transverse plane measurements were inverted. The left cylinder and arrow, which typically represent the left force plate, instead corresponded to the right force plate, and vice versa.

The inter-device variance is summarized in Table 1.

a)

b)

c)

Figure 1 – Examples of software limitation disabling the measurement of horizontal deviations of the most distal force plate in 3D mode (the same accounts for Legacy mode), a) RadboudUMC, b) MRCAardenburg, c) Borrowed L.A.S.A.R.

| **Table 1 – Accuracy measurements per used 3D L.A.S.A.R. device** | | | | | |
| --- | --- | --- | --- | --- | --- |
| **Mode** | | **Force plate** | **Device used** | | |
|  | |  | **RUMC** | **Borrowed L.A.S.A.R.** | **MRCA** |
| **Frontal plane** | **2D** | **Left** | 7mm (-) | 10mm (+) | 0mm |
|  |  | **Right** | 7mm (+) | 7mm (-) | 1mm (+) |
|  | **3D** | **Left** | 4mm (-) | 10mm (+) | 2mm (-) |
|  |  | **Right** | 7mm (+) | 7mm (-) | 1mm (+) |
| **Sagittal plane** | **2D** | **Left** | Not applicable | Not applicable | Not applicable |
|  |  | **Right** | 10mm (+) | 9mm (+) | 3mm (-) |
|  | **3D** | **Left** | Not applicable | Not applicable | Not applicable |
|  |  | **Right** | 8mm towards anterior (right) | 9mm towards anterior (right) | 3mm towards posterior (left) |
| (+): frontal plane – load line is tilted towards the edge, sagittal plane – load line is tilted anteriorly  (-): frontal plane – load line is tilted towards the middle, sagittal plane – load line is tilted posteriorly | | | | | |

From the data of cohort 1, the dorsal and ventral measurements were compared to one another and statistically tested for significant difference. The same was done differentiating between the right force plate of the L.A.S.A.R. (photo perspective) and the left one. It has to be noted that these comparisons (dorsal/ventral perspective and right/left force plates) only apply to the borrowed 3D L.A.S.A.R. device, which was used to measure the validity. The results of the paired-t-tests indicated that the measurement differed significantly on the level of the force plates (right/left) in the frontal plane, whereas the comparison from dorsal to ventral was insignificant, except for the prosthetic leg in 2D mode. In the transverse plane, neither the force plate nor the perspective produced significant bias. Therefore, the validity measures were calculated differentiating on the level of the mode, leg, and force plate.

| **Table 2 – Paired t-tests to determine systemic bias from the different force plates (right vs. left) classified by plane, mode, and leg** | | | | | |
| --- | --- | --- | --- | --- | --- |
|  |  |  | **Mean difference** | **SD difference** | **Significance** |
| **Frontal** | **2D** | **Prosthesis** | 17.65 mm | 10.23 mm | p < .001 |
|  |  | **Intact** | 19.69 mm | 13.40 mm | p < .001 |
|  | **3D** | **Prosthesis** | 16.85 mm | 11.16 mm | p < .001 |
|  |  | **Intact** | 18.85 mm | 10.39 mm | p < .001 |
| **Transverse** | **2D** | **Prosthesis** | -0.13 Nm | 0.89 Nm | p = 0.48 |
|  |  | **Intact** | -0.21 Nm | 1.00 Nm | p = 0.31 |
|  | **3D** | **Prosthesis** | -0.10 Nm | 0.84 Nm | p = 0.56 |
|  |  | **Intact** | -0.11 Nm | 0.78 Nm | p = 0.48 |
| SD: Standard deviation, mm: millimeter, Nm: Newton meter | | | | | |

| **Table 3 – Paired t-tests to determine systemic bias from the different perspectives (dorsal vs. ventral) classified by plane, mode, and leg** | | | | | |
| --- | --- | --- | --- | --- | --- |
|  |  |  | **Mean difference** | **SD difference** | **Significance** |
| **Frontal** | **2D** | **Prosthesis** | 4.89 mm | 20.10 mm | p = 0.02 |
|  |  | **Intact** | 10.92 mm | 21.42 mm | p = 0.23 |
|  | **3D** | **Prosthesis** | 4.62 mm | 19.94 mm | p = 0.25 |
|  |  | **Intact** | 7.85 mm | 20.33 mm | p = 0.06 |
| **Transverse** | **2D** | **Prosthesis** | -0.26 Nm | 0.87 Nm | p = 0.15 |
|  |  | **Intact** | -0.15 Nm | 1.01 Nm | p = 0.46 |
|  | **3D** | **Prosthesis** | -0.16 Nm | 0.83 Nm | p = 0.36 |
|  |  | **Intact** | -0.18 Nm | 0.77 Nm | p = 0.26 |
| SD: Standard deviation, mm: millimeter, Nm: Newton meter | | | | | |
