# Supplementary material for: Reproducibility and validity of the 3D L.A.S.A.R. posture alignment system in persons with a transtibial bone-anchored prosthesis
Source: Prosthet Orthot Int. 2025 Oct 20;50(5):620–9. doi: 10.1097/PXR.0000000000000497 (PMC13645406; doi:10.1097/PXR.0000000000000497)
Supplement: Supplementary file 2 [file poi-50-620-s002.docx]

**Supplemental Digital Content 2 – Transverse plane measurements**

**Introduction**

Deviations in the transverse plane were found to be influential for frontal and sagittal plane measurements. Therefore, the additional question arose if the measurements of transverse plane by the 3D L.A.S.A.R. were also reproducible and valid.

**Method**

All participants followed the standardized instructions as described in the paragraph ‘Study Design’ to acquire a reproducible stance.

The data of the transverse plane measurements were noted from the cylinders and arrows in the bottom corners of the photos from the integrated software by Ottobock.

The statistical analysis was identical to the analysis of the primary parameters and comprised ICC, SEM, SDC and LoA (see article).

**Results**

As the proportion of missing values per variable did not exceed 5%, no imputation was performed. The missing values were considered missing completely at random with an arbitrary missing data pattern

**Reproducibility**

Reliability was found to be very good, with ICC(3,1)_agreement_ ranging from 0.82-0.90 in 2D mode and 0.84-0.91 in 3D mode.

Within the 2D mode the %SEM_agreement_ varied between 22.1%-32.2%, and the %SDC_agreement_ between 60.9%-91.3%. Within the 3D mode, the %SEM_agreement_ varied between 20.9%-26.7%, and the %SDC_agreement_ between 57.4%-74.7% (see Table 4).

| **Table 4 – Reliability and Agreement Parameters of the transverse plane measurements divided per mode, perspective, and leg** | | | | | | | | | | | |
| --- | --- | --- | --- | --- | --- | --- | --- | --- | --- | --- | --- |
|  |  | | **Test scores** | | | **Reliability parameters** | **Agreement parameters** | | | | |
| **Parameter** | | | **Test**  **mean ± SD (Nm)** | **Retest**  **mean ± SD (Nm)** | **Difference (test-retest)**  **mean ± SD (mm)** | **ICC3,1_agreement_ (95%CI)** | **SEM_agreement_ (Nm)** | **%SEM_agreement_** | **SDC_agreement_ (Nm)** | **%SDC_agreement_** | **LoA**  **95%CI (Nm)** |
| **2D mode** | **Ventral** | **Prosthesis** | -1.7 ± 1.7^a^ | -2.0 ± 1.8^a^ | 0.3 ± 0.8^a^ | 0.87^a^ (0.76-0.93)* | 0.6 | 32.2% | 1.7 | 91.3% | -1.3; 1.9 |
|  |  | **Intact** | -1.7 ± 1.3^a^ | -1.9 ± 1.2^a^ | 0.3 ± 0.7^a^ | 0.82^a^ (0.67-0.90)* | 0.4 | 22.1% | 1.1 | 60.9% | -1.1; 1.7 |
|  | **Dorsal** | **Prosthesis** | -1.9 ± 1.7^a^ | -1.8 ± 1.7^a^ | -0.1 ± 0.8^a^ | 0.90^a^ (0.83-0.94)* | 0.5 | 26.5% | 1.4 | 74.3% | -1.7; 1.5 |
|  |  | **Intact** | -1.9 ± 1.5^a^ | -2.0 ± 1.3^a^ | 0.1 ± 0.8^a^ | 0.83^a^ (0.72-0.90)* | 0.6 | 31.5% | 1.7 | 89.1% | -1.5; 1.7 |
| **3D mode** | **Ventral** | **Prosthesis** | -1.7 ± 1.7^a^ | -2.0 ± 1.7^b^ | 0.3 ± 0.8^c^ | 0.89^c^ (0.81-0.94)* | 0.4 | 21.3% | 1.1 | 58.5% | -1.3; 1.9 |
|  |  | **Intact** | -1.7 ± 1.3^a^ | -1.9 ± 1.2^b^ | 0.3 ± 0.7^c^ | 0.85^c^ (0.71-0.92)* | 0.4 | 22.2% | 1.1 | 61.1% | -1.1; 1.7 |
|  | **Dorsal** | **Prosthesis** | -1.9 ± 1.7^a^ | -1.9 ± 1.6^a^ | 0.0 ± 0.7^a^ | 0.91^a^ (0.85-0.95)* | 0.5 | 26.7% | 1.4 | 74.7% | -1.4; 1.4 |
|  |  | **Intact** | -1.8 ± 1.4^a^ | -2.0 ± 1.4 | 0.2 ± 0.8^a^ | 0.84^a^ (0.73-0.91)* | 0.4 | 20.9% | 1.1 | 57.4% | -1.4; 1.8 |
| ^a^ – n=49, ^b^ – n=48, ^c^ – n=47  *p < 0.001  SD: standard deviation, mm: millimeter, ICC: Intraclass correlation coefficient, 95%CI: 95% confidence interval, SEM: Standard Error of Measurement, SDC: Smallest Detectable Change, LoA: Limits of Agreement, Nm: Newton meter | | | | | | | | | | | |

**Validity**

Within the 2D mode, slight to good validity was found with ICC(3,1)_consistency_ varying between 0.29-0.68. Within the 3D mode, validity varied between 0.27-0.63 (see Table 5). The Bland and Altman plots demonstrated that agreement was consistent over the measurement range for all analyses.

| **Table 5 - Validity of the L.A.S.A.R. measurements of the transverse plane compared to Vicon divided per mode, leg and force plate** | | | | | | | | |
| --- | --- | --- | --- | --- | --- | --- | --- | --- |
|  |  |  | **Test scores** | | **Validity scores** | | |  |
|  |  |  | **L.A.S.A.R.** | **Vicon** | **Difference** | **ICC3,1_consistency_** | **LoA** |  |
|  | **Leg** | **Force plate** | **Mean ± SD**  **(Nm)** | **Mean ± SD (Nm)** | **(L.A.S.A.R. -Vicon) Mean ± SD (Nm)** | **(95%CI)** | **95%CI (Nm)** |  |
| **2D mode** | Prosthesis | Right | -1.8 (-2.5; -0.6)^b^ | -3.5 ± 2.5 | 1.6 ± 2.2 | 0.54 (0.20-0.76)* | -2.7; 5.9 |  |
|  |  | Left | -1.6 (-3.1; -0.8)^b,c^ | -3.5 ± 2.5 | 1.7 ± 1.8^c^ | 0.68 (0.39-0.84)^c^** | -1.8; 5.2 |  |
|  | Intact | Right | -2.0 ± 1.5^c^ | -3.7 ± 2.6 | 1.0 (0.2; 3.0)^b,c^ | 0.29 (-0.11-0.61)^c^ | -3.2; 6.6 |  |
|  |  | Left | -1.7 ± 1.3 | -3.7 ± 2.6 | 2.0 ± 2.3 | 0.38 (0.00-0.66)* | -2.5; 6.5 |  |
| **3D mode** | Prosthesis | Right | -1.9 (-2.5; -0.6)^b^ | -3.5 ± 2.5 | 1.6 ± 2.2 | 0.55 (0.22-0.77)* | -2.7; 5.9 |  |
|  |  | Left | -1.4 (-3,0; -0.8)^b,c^ | -3.5 ± 2.5 | 1.7 ± 1.9^c^ | 0.63 (0.32-0.82)^c^** | -2.0; 5.4 |  |
|  | Intact | Right | -1.5 (-3.1; -1.0)^b,c^ | -3.7 ± 2.6 | 1.5 (0.3; 3.0)^b,c^ | 0.27 (-0.13-0.60)^c^ | -3.1; 6.7 |  |
|  |  | Left | -1.7 ± 1.2 | -3.7 ± 2.6 | 1.9 ± 2.3 | 0.33 (-0.05-0.63)* | -2.6; 6.4 |  |
| N.B.: The transverse measurements were derived from photos taken in the frontal perspective solely, excluding the sagittal perspective  SD: standard deviation, Nm: Newton meter, ICC: Intraclass correlation coefficient, 95%CI: 95% confidence interval, LoA: Limits of Agreement  ^b^ - median (25^th^ percentile; 75^th^ percentile) due to non-normal distribution  ^c^ - n=25  * p < 0.05  ** p < 0.001 | | | | | | | |  |

**Discussion**

Test-retest reproducibility and concurrent validity were determined for transverse plane measurements by comparing it with the gold standard Vicon. The results of the transverse plane show the same patterns as in the frontal and sagittal plane. The reliability parameters were high, but the agreement parameters indicate inaccurate measurements, with SEM% starting from 20.9% and SDC% starting from 57.4%.

Concurrent validity was overall lower in the transverse plane than in the frontal or sagittal plane. The ICC’s of the intact leg were significantly lower in the transverse plane. Frontal plane measurements from the same measurement were good compared to slight in the transverse plane (2D), or fair to good compared to slight (3D).
