# Supplementary material for: Reproducibility and validity of the 3D L.A.S.A.R. posture alignment system in persons with a transtibial bone-anchored prosthesis
Source: Prosthet Orthot Int. 2025 Oct 20;50(5):620–9. doi: 10.1097/PXR.0000000000000497 (PMC13645406; doi:10.1097/PXR.0000000000000497)
Supplement: Supplementary file 3 [file poi-50-620-s003.docx]

# **Supplemental Digital Content 3 – Bland and Altman plots (Reproducibility measures)**

Bland-Altman plots for test-retest reproducibility of 3D L.A.S.A.R. measurements of the same participant, classified by mode (2D/3D), plane (frontal/sagittal/transverse), perspective (ventral/dorsal), and leg (prosthesis/intact). (mm) = millimeter, (Nm)= Newton meter; The solid line represents the mean difference (systematic bias) between both measurements, whereas the dashed lines show the 95% limits of agreement (mean_difference_ ± 1.96*SD_difference_).

**2D mode – Frontal**

**2D mode – Sagittal**

**2D mode – Transverse**

**3D mode – Frontal**

**3D mode – Sagittal**

**3D mode – Transverse**
